# Progress on the Integrity Protection in the Natural World Heritage Site and Agroforestry Development in the Buffer Zone: An Implications for the World Heritage Karst

**DOI:** 10.3390/ijerph192416876

**Published:** 2022-12-15

**Authors:** Dong Chen, Kangning Xiong, Juan Zhang

**Affiliations:** 1 School of Karst Science, State Engineering Technology Institute for Karst Desertification Control, Guizhou Normal University, Guiyang 550001, China; 2 School of Management Science, Guizhou University of Finance and Economics, Guiyang 550025, China

**Keywords:** natural world heritage, integrity, agroforestry, buffer zone, implications, world heritage karst

## Abstract

In the face of increasing development pressure, how to fulfill the obligations under the World Cultural and Natural Heritage Convention and maintain the integrity of the Natural World Heritage Site (NWHS) is a major problem to be solved at present. Agroforestry (AF) development in the buffer zone maintains the integrity of NWHS and promotes sustainable ecological and economic development in the buffer zone. Still, few studies on the knowledge system of integrity protection of NWHS and AF development in the buffer zone research have been conducted. To fill this gap, this study conducts a systematic literature review based on 128 related articles retrieved from the Web of Science (WoS) database and the China National Knowledge Infrastructure (CNKI) database. Firstly, quantitative studies were conducted to analyze the annual numbers, content and study regions of the published literature. Secondly, the main research progress and achievements of integrity protection of WNHS and AF development in the buffer zone are classified and summarized. On this basis, this paper proposed key scientific issues that remain to be addressed in future, as well as exploring the implications for the World Heritage (WH) karst. This study is a scientific reference for the balanced development of NWHS integrity protection and AF in the buffer zone.

## 1. Introduction

The Natural World Heritage Site (NWHS) is a natural site or precisely delineated natural area of Outstanding Universal Value (OUV) from the point of view of science, conservation or natural beauty. To become a NWHS, the property must meet one or more of the following criteria: (vii) aesthetics; (viii) geomorphy; (ix) biodiversity; (x) biological habitat [1]. As of 2022, there are 1154 heritages worldwide, including 897 cultural sites, 218 natural sites and 39 mixed sites [2]. NWHS is vulnerable to disasters such as heavy precipitation, floods, fires, earthquakes, landslides, wars [3] and great population pressure. Once destroyed, they are difficult to be replaced or regenerated naturally [4]. Therefore, the NWHS requires special protection against the dangers that increasingly threaten them. The protection of NWHS is based on the principle of integrity, which is not only a yardstick for measuring the value of heritage, but also the key basis to protect the OUV of NWHS [4,5]. However, the concept of heritage protection is no longer limited to the closed protection of the heritage itself, and has shown a trend of outward-looking and regionalized holistic protection [6].

Buffer zones were first formally proposed by Shelford [7], and their primary role was to mitigate the adverse impacts caused of human activities outside the nature reserve area [8]. Notably, as the concept of heritage protection evolves, buffer zones, essential means of comprehensive protection, now receive more attention [9]. This is now widely present in various types of protected areas around the world as one of the most important means of protecting the integrity of the resource [10], and the two types of buffer zone research oriented by ecology and society have gradually transformed into the integration trend of ecological protection and community interests [11]. The buffer zone of WH can not only protect the OUV of heritage sites from external threats, but also enhance the connection with the wider surrounding area and benefit the surrounding communities [12,13,14,15,16].

The buffer zone has a dynamic character as a spatial control method to protect the integrity of heritage sites against external threats. At the same time, buffer zones also threaten heritage site protection [11]. Therefore, in order to ensure the integrity protection of heritage sites and the sustainability of buffer zone development, the harm caused by buffer zone development to heritage sites should be solved through the guidance and adjustment of industrial development, and the industrial structure of buffer zones should not conflict with the protection requirements of heritage sites. AF is a dynamic, ecologically based, natural resource management system that, through the integration of trees in farms and rangeland, diversifies and sustains smallholder production for increased social, economic and environmental benefits [17]. AF in the buffer zone is an ecological industry developed in the form of industry in the buffer zone of NWHS, which can promote the common prosperity of the buffer zone residents and preserve heritage resources. AF development in the buffer zone has the benefits of developing the economy and protecting the environment [18,19,20], especially for karst areas with ecological fragility and a relatively backward regional economy [21]. However, few studies have focused on the integrity protection of NWHS and AF development in the buffer zone. From both theoretical and practical needs, there is an urgent need to implement a more focused and targeted analysis of the relationship between the integrity protection of heritage sites and AF development in the buffer zone, to achieve the goal of balance between the integrity protection of heritage sites and AF development in the buffer zone.

**Figure 1:**
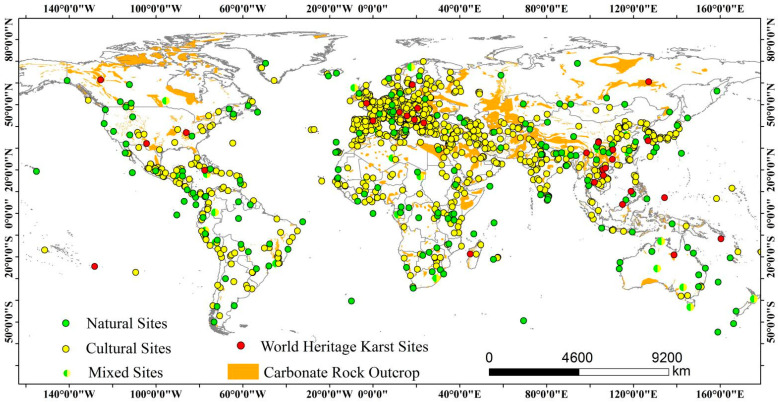
Global distribution of natural sites, cultural sites, mixed sites, and WH karst sites.

Karst is one of the most remarkable landscapes in the world, and it accounts for 12% of the land area [22]. WH karst is a vital part of the NWHS, and there are currently 30 karst sites worldwide, accounting for 13.76% of the NWHS (Figure 1). In karst areas, under the fragile ecological environment, the unreasonable socio-economic activities of human beings have resulted in the prominent contradiction of human–land systems, vegetation degradation, soil erosion, gradual rock exposure, land productivity degradation [23]. Affected by natural conditions and interference from human socio-economic activities, the ecosystem in karst areas is highly fragile and faces numerous ecosystem problems [24]. WH karst ecosystems present complex and fragile features compared to other NWHS ecosystems, and the problem of surging fragile ecological environments with pressing economic development needs is particularly urgent.

**Figure 2:**
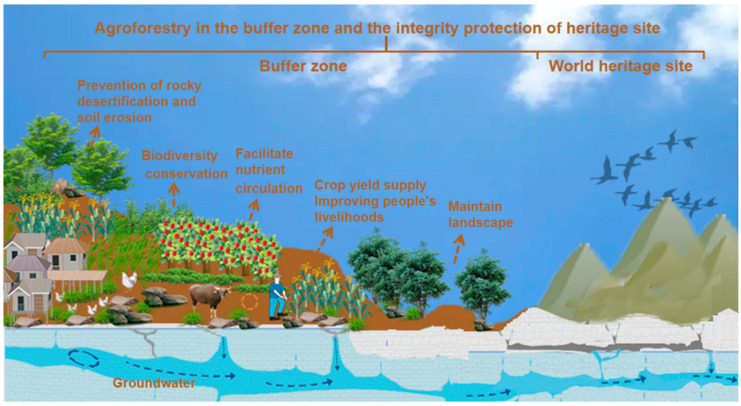
Schematic diagram of the relationship between the integrity protection of NWHS and AF development in the buffer zone.

The special ecological environment and human activities in karst areas have influenced the protection and development of WH karst and even aggravated the vulnerability of karst ecosystems [25]. AF has solid ecological resilience, which can provide opportunities for ecological restoration and improvement of local human livelihoods in ecologically fragile areas [26,27]. AF has achieved excellent results in solving issues related to karst areas in recent years, especially in South China Karst, AF ecological industry models such as forest–fruit, forest–medicine and forest–grass have achieved good ecological, social and economic benefits [28,29]. In addition, AF development in karst areas can achieve the integrity protection of heritage sites and a mutually beneficial relationship between ecological and socioeconomic needs (Figure 2), promoting sustainable region development [30,31,32] and further achieving the dual goals of development and protection [33,34]. At present, most studies on AF in the buffer zone of WH karst have focused on the effects of AF on hydrothermal [35,36], soil nutrients and biodiversity [37,38] and landscape and rural livelihoods [27,39]. Studies have shown that AF has a protective effect of reducing soil erosion, which is an important engineering measure that protects the ecological environment and promotes the socioeconomic development of karst areas [40,41,42]. The development of AF in the buffer zone is important for the integrity protection of heritage sites [18], but there are few relevant studies on the relationship between them. Therefore, there is a need for a comprehensive study of the integrity protection of heritage sites and AF development in the buffer zone. To explore the protection mechanism of AF in the buffer zone on the integrity of NWHS, and to provide scientific reference for heritage protection and sustainable utilization.

To clarify the relationship between the integrity protection of NWHS and AF development in the buffer zone, this paper reviews the research progress and achievements of integrity protection of NWHS and AF development in the buffer zone worldwide. It discusses the intrinsic link between the integrity protection of heritage sites and AF development in the buffer zone, to enlighten the future research direction of WH karst with a fragile ecological environment and promote the sustainable development of the ecological environment and socio-economy in WH karst. To provide a comprehensive and objective overview of the current state of research in the field and provide a scientific reference for subsequent research. To achieve this, the framework of this paper is as follows:

Section 3: the essential characteristics of the literature (year variation of the literature, research content, research area).

Section 4 and Section 5: the key areas of research progress (current research progress, landmark achievements, key scientific issues to be addressed in future, and implications for WH karst).

Section 6: conclusion and future research (the future research directions for the integrity protection of WH karst and AF development in the buffer zone).

## 2. Methods

### 2.1. Literatures Acquisition Sources

To identify relevant studies, this search was conducted based on the platforms including WoS (https://www.webofscience.com, accessed on 1 July 2021) and CNKI (https://www.cnki.net/, accessed on 1 July 2021). The WoS is a large comprehensive, multidisciplinary, core journal citation index database. CNKI has the largest database in China, including all published papers and literature. These databases can help us to target current research hotspots to search, which is why we selected them. The search process was as follows (Table 1). 

We initially searched based on specific keywords and found 281 records, of which 179 articles were in English and 102 articles were in Chinese (Table S1). The time range of the search was the maximum time range of both databases, and the search time was up to 30 June 2022.

### 2.2. Literatures Selection Criteria

**Figure 3:**
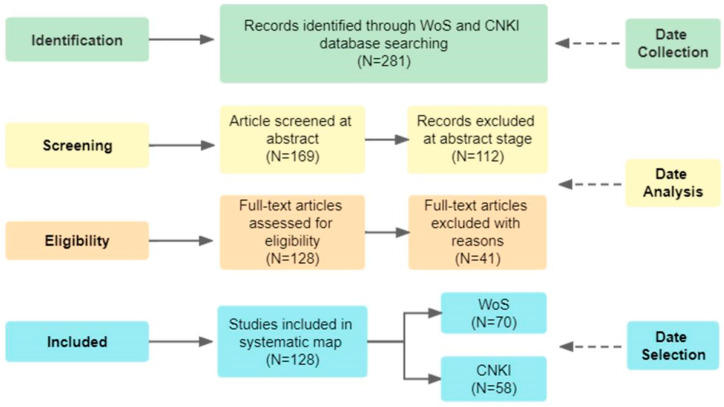
Data selection process.

According to the research content of this paper, the literature related to the research content were screened out by the titles, abstracts, keywords and full-text articles in turn, and the duplicate and irrelevant literature were eliminated manually. Finally, 128 relevant papers were identified. This included 114 journal papers, 3 Ph.D. theses, 6 masters theses, 4 reviews and 1 conference paper, of which 70 articles were in English and 58 articles in Chinese (Figure 3). 

## 3. Results

### 3.1. Annual Distribution of the Literature

#### 3.1.1. The Infancy Phase

**Figure 4:**
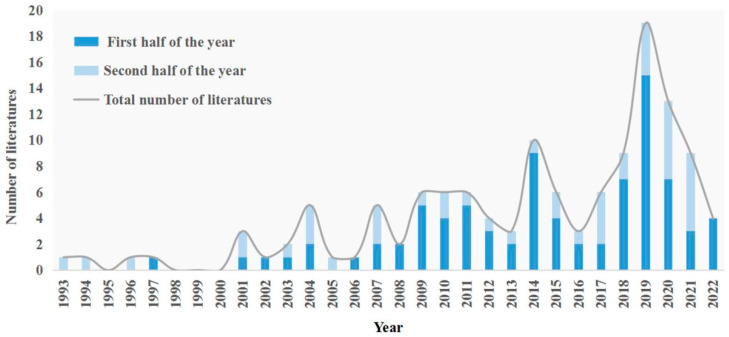
Annual distribution of the literature.

The annual distribution of research literature on the integrity protection of NWHS and AF development in the buffer zone can be broadly divided into three phases (Figure 4). The first phase, from 1993 to 2008, when the buffer zone was only a tool recommended to States Parties by the World Heritage Conservation Organization, so with no more than five studies per year, indicates that this phase was still in its infancy. 

#### 3.1.2. The Fluctuating Growth Phase

Since the discussion on the application of the buffer zone in practice at the Davos International Conference in 2008, there has been a profound change in heritage protection methods and perceptions. The increasing number of problems faced when using the buffer zone to conserve heritage has prompted more scholars to focus on the impact of buffer development on heritage site protection, and the research on them in heritage protection has gradually shown intensiveness. As a result, from 2008 to 2016 was fluctuating growth.

#### 3.1.3. The Rapid Growth Phase

After the fluctuating growth in 2008–2016, the next period of rapid growth occurred in 2016–2022. With the increasing conflict between the protection and development of NWHS in the world, the issue of NWHS integrity has come to prominence. How to assess the harmony between heritage sites and buffer zones has gradually received researchers’ attention. Overall, the number of studies on the integrity protection of NWHS and AF development in the buffer zone has fluctuated, with the highest number of publications in 2019. It shows a general increase in research on the integrity protection of NWHS and AF development in the buffer zone, which is relatively new. In addition, the number of literature published in the first half of the year was nearly twice that of the second half. 

### 3.2. Content Distribution of the Literature

**Figure 5:**
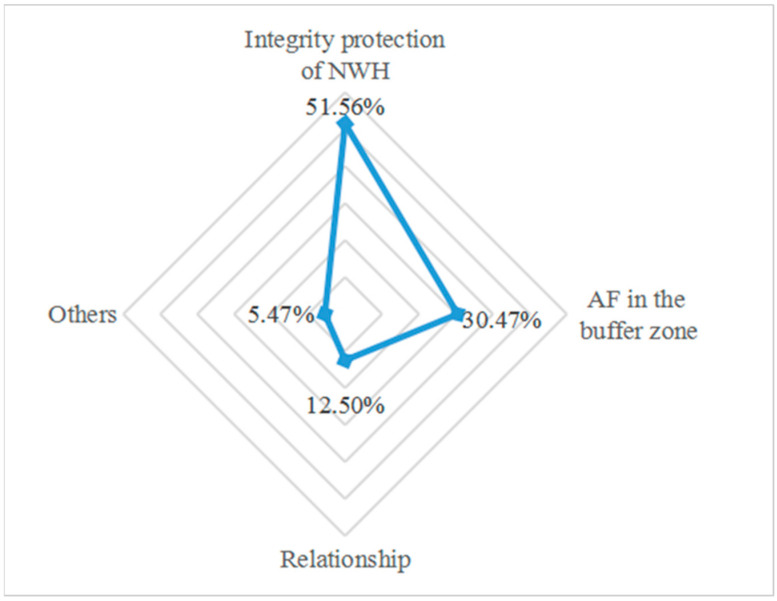
Distribution of research contents.

After reviewing 128 different papers, we classified and summarized the literature into the integrity protection of NWHS, AF in the buffer zone, the relationship between the two and other types of studies (Figure 5). Among them, the integrity protection of NWHS documents account for 51.56% of the total, AF in the buffer zone documents accounts for 30.47%, the relationship between integrity protection of NWHS and AF in the buffer zone accounts for 12.50%, and other types of documents account for 5.47%. It indicated that the existing studies were more focused on the integrity protection of NWHS and less on the relationship between the integrity protection of NWHS and AF in the buffer zone.

### 3.3. Distribution of Literature Study Areas

**Figure 6:**
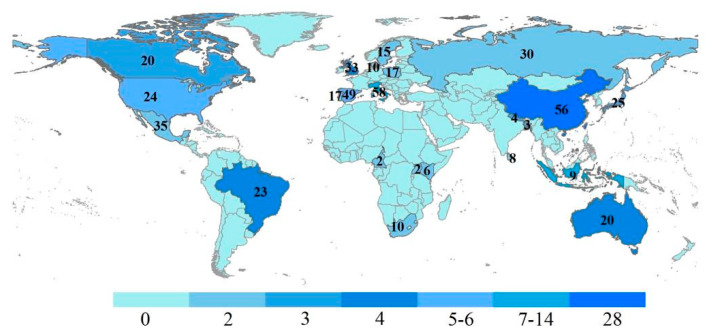
Research regions distribution of literatures and the number of WH owned by the States Parties.

Based on the analysis of the literature, a statistical analysis of the global distribution of research regions of the 128 obtained papers were conducted (Figure 6). The research areas are mainly distributed in China, Italy, Indonesia, the United States, Spain and other countries. South China Karst, Tianshan of Xinjiang, Tunisia, Bromo Tengger Semeru and other regions are the hotspot areas for the integrity protection of heritage sites and AF in the buffer zone [18,43,44,45]. In addition, in terms of the regional distribution of the study, countries such as China and Italy produced the highest number of publications, which may be due to the two States’ Parties ranked among the highest number of heritage owned in the world. Moreover, studies on AF development in the buffer zone were first conducted in Gunung Palung National Park, West Kalimantan, Indonesia [46].

## 4. Main Progress and Landmark Achievements

### 4.1. Integrity Protection of NWHS

#### 4.1.1. The Concept of Integrity

The word “integrity” is derived from the Latin root and has two meanings, one is secure and the other is complete and total [47]. From a historical perspective, integrity means the condition of minimal influence. In modern language, it is generally understood to be intact and original [48]. Moreover, the principle of integrity is an important guiding principle for the protection of NWHS and is fundamental to ensuring the sustainability of heritage sites, ensuring the value of WH while also delineating the principle scope for their protection [49,50]. Combining the Operational Guidelines for the Implementation of the World Heritage Convention (WHC) (hereinafter referred to as the Operational Guidelines) with the related studies on integrity, the following developmental changes in the concept of integrity are summarized (Table 2).

The concept of integrity remains unchanged from the 2008 to the 2021 version, i.e., all properties nominated for inscription on the World Heritage List (WHL) shall satisfy the conditions of integrity. Integrity measures the wholeness and intactness of the natural and/or cultural heritage and its attributes. Examining the conditions of integrity, therefore, requires assessing the extent to which the property: (a) includes all elements necessary to express its OUV; (b) is of adequate size to ensure the complete representation of the features and processes which convey the property’s significance; (c) suffers from adverse effects of development and/or neglect. This should be presented in a statement of integrity [1]. Therefore, combined with the description of the concept of integrity in the Operation Guide, the concept of integrity can be roughly defined as the maintenance and protection of the integrity of the NWHS and its characteristics, the state of absence of defects and the significance of inheritance.

Integrity, however, is an elusive concept for which the United Nations Educational Scientific and Cultural Organization (UNESCO) provides no definitional protocol, and for which the scientific community objects to a static or pure interpretation, how to scientifically evaluate the integrity of NWHS is the key to the protection and development of NWHS [51,52], and further research is necessary. Karst is a geomorphology formed from the corrosion, precipitation, erosion, and sedimentation of soluble rocks in response to underground water and surface water flow and gravitational collapse, subsidence, and stacking [53]. Karst areas are characterized by diverse karst landforms, vulnerable ecosystems, considerable karst desertification and recurrent geological disasters [54,55]. Because of their special geological formations, karst areas can nurture spectacular landform types and stunning landscapes instead. Given the fragility of these environments and the complex interconnectedness of karst landscape elements, it is critical to evaluate the integrity protection of heritage sites. Nevertheless, to effectively assess the WH karst integrity requires more exploration. In particular, it is crucial to establish a system of integrity assessment indicators based on karst heritage site characteristics as a guide.

#### 4.1.2. Integrity of Aesthetic Values

All properties nominated for inscription on the WHL shall satisfy the condition of integrity, outstanding landscape is the core embodiment of the aesthetic value of a NWHS [56]. When identifying the uniqueness of various landscapes, their relevance in geographical space and ecological processes cannot be ignored [57]. In the quantification of integrity, the study area was spatially expressed according to the unique landscape types which are of adequate size to ensure the complete representation of the landscape’s aesthetic features and significance [58]. Moreover, unlike the general scenic spots, the landscape aesthetic value of heritage sites has its particularity. Thus, the degree of uniqueness of landscape types should be considered in landscape aesthetic value assessment. Although landscape preferences and beauty have subjective elements, it is significant and worthwhile to obtain ways of assessing objectively the integrity of landscapes.

With the development of RS and GIS, some researchers have begun to discuss the application of these technologies in assessing the landscape and its protection [59,60]. The application of GIS has gradually shifted landscape assessment from qualitative to quantitative. Han [56] employed a visual index of outstanding landscape, a harmony index of the artificial landscape and a disturbance index of damaged landscape to measure the impact of landscape integrity through viewshed analysis using ArcGIS. Ha and Yang [58] established a universal system based on ArcGIS combining subjectivity and objectivity to evaluate the landscape aesthetic value of a NWHS. In the indicator of NWHS criteria, integrity was considered the most significant factor to influence. Xiao [61] used GIS technology to evaluate the impact of the Zhangjiajie Grand Canyon Glass Bridge on the WH value of Wulingyuan by using the view analysis method. The results showed that its heritage site aesthetic value and integrity were not affected. In addition, researchers also constructed evaluation indexes for the integrity of heritage sites and national parks from the aspects of ecosystem component integrity, ecosystem structural integrity, ecosystem functional integrity and landscape composition integrity, such as landscape type function dominance, degradation of landscape type and landscape fragmentation [43,62].

Despite the close human dependence on karst, conservation policies are ineffective on a landscape scale. As karst systems are intrinsically fragile environments with high connectivity among their elements, they would benefit from a landscape protection approach that goes beyond the conservation of single caves or single cave species [63]. Karst is one of the most distinguished landscapes in the world. When evaluating the aesthetic value of the WH karst landscape, the uniqueness of the WH karst landscape should be completely taken into account with RS and GIS techniques, and an objective evaluation system should be established by exploring evaluation methods suitable for assessing the aesthetic value of the WH karst landscape.

#### 4.1.3. Integrity of Geomorphic Values

NWHS is a complex of geomorphologic and biological elements worthy of protection [64,65]. The WHL now includes 1154 sites of OUV, 93 of which are recognized, in part or full, for their geological values. This heritage includes the most exceptional places that record the geological history of planet earth, life and evolution, and the physical processes that shape our landscapes [66]. Among them, the geological and geomorphological value criteria are prominent examples of important stages in the earth’s evolutionary history, including the earth’s history, life record, important geological processes in landscape evolution and significant geological or geomorphological features [67]. Within this general context, other aspects of integrity are mostly specific to the theme and property under consideration [68]. Distinctive geological, geomorphological and ecological components and their mutual interactions of heritages should be paid to geological and ecological connectivity to protect the integrity of natural heritage [69].

Due to their integrity, the majority of geomorphosites have a relatively high scientific value [70]. Furthermore, the landscape value of a NWHS includes its scientific importance as well as social, economic and landscape aspects [71]. Most of them have relatively high scientific, ecological and aesthetic value due to their integrity, representativeness and rarity, of which geomorphic landscapes are a central component. The intrinsic value of geomorphological landscapes is considered in the context of aesthetic, cultural, socio-economics and ecology and geoscience [72]. Moreover, the geomorphic landscape is one of the OUV of NWHS and an essential resource for developing the tourism economy, which is receiving increasing attention in various aspects such as tourism, regional development and resource protection.

Karst landscapes are mainly developed in carbonate rock areas and a special type of landform formed by soluble rocks under the joint action of surface and groundwater and internal and external forces of the earth, which generally have strong ornamental, artistic and scientific values [73]. Current karst landscape evaluations face the problems of a single method, evaluation subject and evaluation type [74]. Therefore, the scientific, ecological and aesthetic values of karst landscapes and the impact of their unique features on the integrity of WH karst landscapes should be thoroughly considered, and a comprehensive rating index system suitable for the integrity of WH karst landscapes should be explored.

### 4.2. The Relationship between Integrity Protection of NWHS and AF in the Buffer Zone

#### 4.2.1. AF in the Buffer Zone Can Achieve the Goal of Integrity Protection of NWHS

Zhang [75] argued that economic growth should be transformed in the buffer zone of WH karst by establishing a highly productive and sustainable AF model, thus strengthening the socio-economic functions of the buffer zone. AF is a collective name for land-use systems and technologies where woody perennials are deliberately used on the same land-management units as agricultural crops and/or animals, which can create environmental, economic and social benefits [76,77,78]. It also can advance regenerative agriculture’s core environmental concerns: soil fertility and health, water quality, biodiversity, ecosystem health and carbon sequestration [79,80,81]. In conclusion, the AF system is a land-use practice with high levels of productivity, sustainability and adoptability; among them, sustainability is the biggest advantage of AF [82,83]. The buffer zone gives an added layer of protection to the heritage site and it has two main functions: ecological buffer and resource protection, community development and coordination [1,12]. Construct AF ecosystems in ecological transition zones can play a two-way ecological buffer function [84]. Enhancing AF development in the buffer zone can reduce the need to harvest resources from protected areas [85,86,87], while improving rural livelihoods and economic benefits [88,89]. Moreover, the value of AF systems can be presented through tourism and local activities [90].

The integrity protection of NWHS and AF development in the buffer zone have a mutually reinforcing and coordinated relationship. AF in the buffer zone is a common goal to enhance the economic development of local residents and reduce the dual pressure of population growth, and ensure the integrity of heritage sites is protected. For NWHS, transition and cohesion are the key to the spatial coordination of buffer zones, which should not only emphasize the protection function of buffer zones one-sidedly, but also focus on the negative impacts of buffer zone settings on residents, and seek multiple ways to benefit residents and communities by allowing appropriate economic activities and management policies. This way, policy implementation and protection management of buffer zones can be supported to promote the connection between heritage sites and the wider surrounding area and the sustainable development of surrounding communities [11,91]. As for the buffer zone, heritages site can effectively develop and protect valuable natural scenery or geological relics, alleviate ecological and environmental problems and serve as platforms for popular science education and scientific research. For a region in particular, it means infinite development potential and broad economic prospects.

#### 4.2.2. AF in the Buffer Zone Promotes the Sustainability of WH Landscapes

In part, the integrity of a NWHS depends on the connection of the wider landscape [92]. The buffer zone help protect the integrity and sustainability of a heritage site. AF as a land-use and landscape concept constantly evolves, putting landscapes and livelihoods (i.e., the landscape level) into the foreground [93,94]. Landscapes, where AF is practiced, contribute to the full range of goods and services from agriculture to natural resources needed for social and ecological sustainability [95,96]. AF has a wide range of environmental, social, cultural and economic benefits at the landscape scale [97,98]. Attwater and Merson [99] argued that agriculture has an essential landscape advantage and buffer role between Sydney’s urban sprawl and WH due to urban growth and increased land values. Vallejo [100] implemented a multi-scale management strategy for traditional AF as a rural landscape with high cultural relevance in community areas and regions. Previous studies indicated that AF diversity can improve landscape sustainability [101].

AF in the buffer zone of WH karst should give adequate consideration to its economic and ecological benefits and give fair play to its scenic advantages and buffer role in protecting the integrity of heritage sites. While natural heritage landscapes are of the highest natural phenomena or extraordinary beauty, landscapes are also among the most fragile and sensitive properties of natural heritage. In response to the fragility, complexity and uniqueness of different karst landscapes, a proper understanding and evaluation of the karst landscape is crucial for its protection [102,103]. Amaral [104] evaluated the anthropic effects on the landscape structure of the Lencois Maranhenses National Park and its buffer zone, and proposed the implementation of AF systems with agro-successional restoration goals recommended as an alternative for land use. Moreover, it has been proven that AF has become a vital development objective to protect the karst ecological landscape [27]. It is critical to include social perceptions of the landscape and to manage the tradeoff between human activity and karst conservation to ensure both are preserved. Which can strengthen relationships between populations and their surroundings to underpin sustainable development [105].

#### 4.2.3. AF in the Buffer Zone Can Maintain the Geomorphological Integrity of the Heritage Site

Studies have shown that AF played a role in combatting deforestation and preventing erosion, combatting desertification and drought, supporting sustainable development in mountainous areas and promoting rural sustainable development [106,107,108]. WH geomorphologies tend to be of high conservation value due to their distinct topographic features and ecological environments. AF can provide a wide range of ecosystem services, improve soil productivity, enhance erosion control and increase water availability [87,109,110].

It is necessary to protect the fragile landforms of WH karst by developing AF. Simultaneously, approximately 17% of the human population lives in karst areas, and 25% of them rely on groundwater [111,112], making these areas particularly valuable. The significant contradiction between people and land is exceptionally outstanding in the karst region due to the thin soil and low productivity of karst ecosystems and the rapid population [113]. As a vast degraded land ecosystem, the karst region is currently experiencing serious conflicts between the restoration of degraded vegetation communities and agricultural activities [114]. Furthermore, the adverse impacts intensified as water consumption increased because of population growth and the absence of continuous surface water supply from porous soil [103,115,116]. Ribeiro and Zorn [117] according to the qualitative analysis, showed that agriculture positively affected the sustainable development of karst landform. Soil and water conservation and ecological benefits in karst areas can be ensured through the development of AF [40].

## 5. Key Scientific Issues to Be Solved

### 5.1. Given the Lag in Applying Buffer Zone Theory in AF Practice, Strengthen the Value of Buffer Zones and Evaluate the Threat Factors of Heritage Site

The buffer zone is a zoning management approach to reduce external threats to heritage boundaries (especially human activities), and protect and enhance the OUV of WH. They can increase the level of contact between the WH and surrounding communities, including indigenous people living within the buffer zone, and should be resilient and flexible in their use [11]. Currently, most research on the buffer zone is at the theoretical stage, and most scientific research and monitoring are centered on the heritage itself. There is a lack of attention to the surrounding areas of buffer zones, and studies on the identification and evaluation of buffer zone values and threat factors are even less common. 3S technology is a collective term for remote sensing (RS), geography information systems (GIS) and global positioning systems (GPS). It is one of the popular technologies for information technology applications now, and is also a comprehensive information technology tool to achieve dynamic acquisition, editing and processing, storage and management, analysis and mining and the application expression of regional information [118,119]. Therefore, in the future, we should focus more on specific values and use 3S technology to implement carrier and carrier feature identification, as well as quantitative evaluation and data for the regions involved in the values. In particular, for WH karst with fragile ecosystems, there is an urgent need to identify and assess the value of buffer zone and threats to heritage sites.

### 5.2. Considering the Difficulty in Quantifying the Integrity Evaluation Index of WH Karst, a Systematic and Scientific Evaluation Index System Is Constructed through Integrity Connotation and 3S Technology

The evaluation system and indicators for the integrity of NWHS are still in the development stage and no clear and consensus evaluation system has been formed. Therefore, it is necessary to first clarify the basic principles of evaluation system construction from scientific connotations and operational guidelines on the three dimensions of integrity. The integrity of NWHS covers the surrounding spatial area closely related to the natural heritage. Integrity requires that OUV elements and areas with heritage values have uncompromising and regional holistic characteristics [120,121]. When compared to other landscape styles and ecosystems, karst has a number of unusual characteristics that must be taken into account when its integrity is assessed: (1) karst is complex; (2) karst ecosystems are fragile; (3) karst integrity depends above all on hydrological conditions [122]. For WH karst, the identification and construction of integrity indicators can be based on the recognition and protection of OUV, and it is imperative to construct an evaluation indicator system for the integrity of WH karst with OUV as the core, using 3S technology, starting from the categories, composition indicators and influencing factors of WH karst integrity. The evaluation results can provide a scientific basis for the integrity protection of the WH karst.

### 5.3. Because of the Unbalance of Ecological–Social–Economic Development of AF in the Buffer Zone of WH Karst, Strengthen the Evaluation Study of AF Sustainable Development

**Figure 7:**
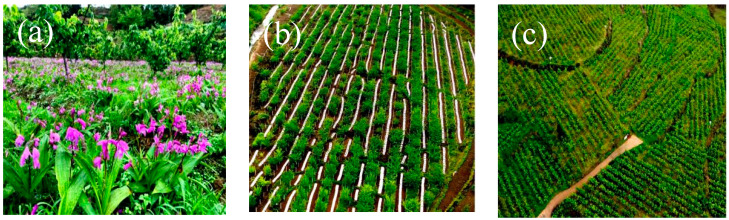
Typical AF in the buffer zone of WH karst ((**a**): Pear + Bletilla; (**b**): Plum + Passion fruit; (**c**): Pear + Miltiorrhiza).

AF is increasingly being recognized as a holistic food production system that can have numerous significant environmental, economic and social benefits [123]. The results from Sun [124] demonstrated that achieving economic development and environmental protection is possible through the implementation of sustainable AF systems in sub-tropical regions. Meanwhile, the economic and social aspects of AF were regularly ignored in assessing its sustainability [125]. Most researchers usually solely concentrate on environmental benefits, while farmers are only concerned about the immediate economic benefits and profits [83]. Therefore, it is necessary to evaluate the sustainability of AF by analyzing the balance between the environment and economy to achieve the socioeconomic and environmental goals of the community. The application of comprehensive index evaluation methods, and the ecological, social and economic development of AF is comprehensively evaluated with multidisciplinary synthesis, multi-angle analysis and multi-method integration to fundamentally solve the threat factors of AF development in the buffer zone and integrity protection of heritage site, and provide a scientific reference for the balanced development of integrity protection heritage site and buffer zone eco-industry. AF provides many ecosystem services and environmental benefits [126], but these may vary by ecological and economic region. Therefore, the WH karst should analyze the ecological–social–economic benefits of karst AF development based on understanding the impact of karst binary structure on AF development, selecting the most representative metrics to assess its sustainability (Figure 7a).

### 5.4. Aiming at the Coupling Relationship between the Integrity Protection of WH Karst and AF Development in the Buffer Zone, and In-Depth Research on Evaluating Indicators, Clarifying the Mechanism and Relation between Them through Qualitative and Quantitative Analysis

Quantifying and clarifying the coupling mechanism between the integrity protection of NWHS and AF development in the buffer zone is of great significance. The buffer zone provides ecological, social and economic benefits in supporting the integrity of protected areas [127], Zhang [75] argued that developed resilient agricultural planting techniques that adapt to environmental changes in the buffer zone, build high-yielding and sustainable AF models, and explore their role in revitalizing eco-industries (Figure 7b). AF’s greatest advantage is ecological sustainability [108], using the AF model, economic development goals and the ecological environment of the communities around the nature reserves can be maximized without damage [128]. The mechanism of integrity protection of WH karst and AF development in the buffer zone is still unclear. The key to the integrity protection of WH karst is to clarify the impact of AF on the heritage site thoroughly. Consequently, it is necessary to establish a set of indicators to assess the integrity protection of WH karst and to monitor the dynamic changes in the development of AF in real time. In addition, for a NWHS, the integrity of NWHS is not static integrity but dynamic, sustainable integrity [129]. Establishing systematic and scientific evaluation indicators will help timely make scientific protection decisions, adjust the development pattern of AF in the buffer zone (Figure 7c), protect the integrity of a heritage site, and achieve a balance between the protection of a heritage site and AF development in the buffer zone.

## 6. Conclusions

In this paper, we performed a systematic literature review by analyzing 128 articles retrieved from the WoS and CNKI. The main conclusions are as follows: (1) studies on the integrity protection of NWHS and AF development in the buffer zone are increasing rapidly, showing a broad research prospect, research regions mainly in China and Italy; Foreign research on AF development in the buffer zone predates domestic research; (2) among the studies on the integrity protection of NWHS, AF in the buffer zone, and the relationship between the integrity protection of NWHS and AF in the buffer zone, the studies on the integrity protection of NWHS are most common, mainly focusing on the impact of NWHS development on integrity; (3) the future focus is to explore on the relationship between the integrity protection of NWHS and AF in the buffer zone. Studies on evaluating the integrity of NWHS should focus on all or some of the criteria (vii) to (x) for the inscription of NWHS on the WHL. In contrast, studies of AF in the buffer zone can focus on its sustainability.

In conclusion, this paper has summarized the current main research progress and achievements in NWHS and AF development in the buffer zone, including the integrity protection of NWHS, AF in the buffer zone and the relationship between the integrity protection of NWHS and AF development in the buffer zone. Key scientific issues that need to be addressed within the scope of this research topic are discussed and future research directions are indicated for protecting the integrity of the WH karst and the development of AF in the buffer zone.

The future directions of integrity protection of WH karst and AF development in the buffer zone can be conducted based on the following aspects: evaluate the value of the buffer zone and threat factors to the heritage site based on the identification and quantification of buffer value and carrier characteristics; construct a systematic and scientific evaluation index for integrity protection of WH karst based on the connotation of integrity and 3S technology and the integrity evaluation indexes of other heritage sites and national parks; improve the sustainability of AF development based on the ecological–social–economic benefits of AF development in the WH karst buffer zone; establish a coupling coordination model to elucidate the relationship and mechanism between them, based on the evaluation indexes of heritage site integrity protection and AF development in the buffer zone. In addition, the issues of integrity protection of WH karst and AF development in the buffer zone have not yet been discussed in depth, and research on the setting of evaluation indexes, the interrelationship among evaluation indicators and specific suggestions for protection and management need to be further developed.

## Figures and Tables

**Table 1:** Literatures search strings.

Database	Retrieval String	Number	Search Date
Wos	First search string: “natural world heritage”	66	30 June 2022
	second search string: “integrity protection”		
	First search string: “buffer zone”	99	30 June 2022
	Second search string: “agroforestry”		
	First search string: “natural heritage”	14	30 June 2022
	Second search string: “agroforestry”		
CNKI	“the integrity of natural heritage”	100	30 June 2022
	“agroforestry in the buffer zone”	1	30 June 2022
	First search string: “natural heritage”	1	30 June 2022
	Second search string: “agroforestry”		
Total		281	30 June 2022

**Table 2:** Integrity concept changes.

Year	Integrity Concept Changes
1977	The concept of integrity first appeared, and the perception of the concept was limited to the ecological soundness of the system, focusing on the constituent elements of heritage in the physical space
1980	Increased integrity conditions for migratory species requiring habitat protection throughout their life cycle
1983	An addition to the integrity requirement: in the case of migratory species, seasonal lots are necessary for the survival of the species and the lot should be given adequate protection regardless of where the species is located
1988	Added an article to the original; the nominated site should contain adequate long-term legislative, regulatory and institutional protections, further focusing on whether the physical element is in good condition
1994	The concept of integrity was initially developed, adding to previous ones the importance of management planning, local legislation, local conservation management bodies and nominated site boundaries, while more specific requirements for the protection of biodiversity in heritage sites were introduced in response to the new global convention on biodiversity
1996	Emphasize that natural aesthetics criteria should not be declared as separate criteria (except in special cases)
1997	Emphasize that the (iv) criterion must include the habitat conditions necessary for the survival of the species
1998	The completeness condition is explicitly proposed to be applied to each criterion
1999	Establishes the importance of local traditional protection methods for integrity
2005	The concept of integrity includes two elements: wholeness and intactness, and examples of the application of the integrity condition to declared properties based on criteria (i) to (vi) are being developed.
2008–2021	All properties nominated under criteria (vii)–(x), bio-physical processes and landform features should be relatively intact, and each criterion has a corresponding condition of completeness

## Data Availability

Data sharing is not applicable to this manuscript as no datasets were generated or analyzed.

## Supplementary Materials

The following supporting information can be downloaded at: https://www.mdpi.com/article/10.3390/ijerph192416876/s1, Table S1: List of the 128 publications considered in bibliometric analysis.
