# Supplementary material for: Progress on the Integrity Protection in the Natural World Heritage Site and Agroforestry Development in the Buffer Zone: An Implications for the World Heritage Karst
Source: Int J Environ Res Public Health. 2022 Dec 15;19(24):16876. doi: 10.3390/ijerph192416876 (PMC9779685; doi:10.3390/ijerph192416876)
Supplement: Supplementary file 1 [file ijerph-19-16876-s001.zip › ijerph-2072448-supplementary.pdf]

Table S1. List of the 128 publications considered in bibliometric analysis

Authors	Year	Name of article	Publication
Luo, X., Xiong, K., Zhang, J., Chen, D.	2021	A study on optimal agroforestry planting patterns in the buffer zone of world natural heritage sites	Sustainability
Galvão,C.A.,Francisco,M.R.,Schlindwein , M.N.	2018	Agroforestry system as a buffer zone in a Brazilian Atlantic Forest conservation unit: an artificial nest predation perspective	Biota Neotropica
Rahman, H.M.T., Deb, J.C., Hickey, G.M.	2014	Contrasting the financial efficiency of agroforestry practices in buffer zone management of Madhupur National Park, Bangladesh	Journal of forest research
Kasolo, W.K., Temu, A.B.	2008	Tree species selection for buffer zone agroforestry: the case of Budongo Forest in Uganda	International Forestry Review
Naughton-Treves, L., Salafsky, N.	2004	Wildlife conservation in agroforestry buffer zones: opportunities and conflict	Agroforestry and biodiversity conservation in tropical landscapes
Salafsky, N.	1993	Mammalian use of a buffer zone agroforestry system bordering Gunung Palung National Park, West Kalimantan, Indonesia	Conservation Biology
Rahman, M.M., Vacik, H., Begum, F.	2007	Comparison of structural diversity of tree-crop associations in Peripheral and Buffer zones of Gachabari Sal forest area, Bangladesh	Journal of Forestry Research
Aumeeruddy, Y., Sansonnens, B.	1994	Shifting from simple to complex agroforestry systems: an example for buffer zone management from Kerinci (Sumatra, Indonesia)	Agroforestry Systems
Kholilah, T.N., Rahimah, H., Hamzah, R.	2019	The sloping agricultural land management on buffer zone of Bromo Tengger Semeru National Park in Malang Regency	IOP Conference Series: Earth and Environmental Science.
Mukadasi, B., Kaboggoza, J.R., Nabalegwa, M.	2007	Agroforestry practices in the buffer zone area of Mt Elgon National Park, eastern Uganda	African journal of ecology
Suyadi, S., Sumardjo, S., Uchrowi, Z.	2019	Factors affecting agroforestry farmers' capacity surrounding national park	Indonesian Journal of Forestry Research
Nyhus, P., Tilson, R.	2004	Agroforestry, elephants, and tigers: balancing conservation theory and practice in human-dominated landscapes of Southeast Asia	Agriculture, ecosystems & environment
Götmark, F., Söderlundh, H., Thorell, M.	2000	Buffer zones for forest reserves: opinions of land owners and conservation value of their forest around nature reserves in southern Sweden	Biodiversity & Conservation
Nadhira, S., Basuni, S.	2021	Implementation of the Concept of Conservation Area Buffer Zone in Indonesia	Journal of Tropical Forest Management
Chowdhury, S., Koike, M.	2010	Therapeutic use of plants by local communities in and around Rema-Kalenga Wildlife Sanctuary: implications for protected area management in Bangladesh	Agroforestry Systems

Table S1. List of the 128 publications considered in bibliometric analysis

Authors	Year	Name of article	Publication
Amaral, Y.T., dos Santos, E.M., Ribeiro, M.C., Barreto, L.	2019	Landscape structural analysis of the Lencois Maranhenses national park: implications for conservation	Journal for nature conservation
Atsri, H.K., Abotsi, K.E., Kokou, K., Dendi, D., Segniagbeto, G.H., Fa, J.E., Luiselli, L.	2020	Ecological challenges for the buffer zone management of a West African National Park	Journal of Environmental Planning and Management
Mehta, N.G., Leuschner, W.A.	1997	Financial and economic analyses of agroforestry systems and a commercial timber plantation in the La Amistad Biosphere Reserve, Costa Rica	Agroforestry Systems
Murniati, Garrity, D.P., Gintings, A.N.	2001	The contribution of agroforestry systems to reducing farmers' dependence on the resources of adjacent national parks: a case study from Sumatra, Indonesia	Agroforestry Systems
Reyes, T., Quiroz, R., Msikula, S.	2005	Socio-economic comparison between traditional and improved cultivation methods in agroforestry systems, East Usambara Mountains, Tanzania	Environment management
Straede, S., Treue, T.	2006	Beyond buffer zone protection: A comparative study of park and buffer zone products' importance to villagers living inside Royal Chitwan National Park and to villagers living in its buffer zone	Journal of environment management
Edirisinghe, G., Surasinghe, T., Karunarathna, S.	2018	Chiropteran diversity in the peripheral areas of the Maduru-Oya National Park in Sri Lanka: insights for conservation and management	Zookeys
Amaral, Y.T., dos Santos, E.M., Barreto, L.	2019	Landscape structural analysis of the Lencois Maranhenses national park: implications for conservation	Journal for nature conservation
Cerqueira, M.C., Matricardi, E.A.T., de Oliveira, C.H.	2021	Landscape fragmentation in a buffer zone and the Nascentes das Geraizeiras Sustainable Development Reserve, Minas Gerais	Ciencia florestal
Khalaf, R.W.	2020	The Implementation of the UNESCO World Heritage Convention: Continuity and Compatibility as Qualifying Conditions of Integrity	Heritage
Kormos, C.F., Bertzky, B., Watson, J.E.M.	2016	A Wilderness Approach under the World Heritage Convention	Conservation letters
	2011	Impact Assessment and Protection of Outstanding Landscape Integrity in a Natural Heritage Site: Fairy Valley, Kanas Nature Reserve, Xinjiang, China	Journal of mountain science
Chakraborty, A., Takenaka, T.	2019	A Qualitative Exploratory Analysis of Ecological Integrity for Safeguarding World Natural Heritage Sites: Case Study of Shiretoko Peninsula, Japan	Heritage

Table S1. List of the 128 publications considered in bibliometric analysis

Authors	Year	Name of article	Publication
Gullino, P., Larcher, F.	2003	Integrity in UNESCO World Heritage Sites. A comparative study for rural landscapes	Journal of cultural heritage
Chen, Y.H., Liu, Q.	2012	The value system and its protection and utilization of natural and cultural heritage in China	Geographical Research
Hu, X.D., Hu, K.H., Zhang, X.P., Tang, J.B.	2019	Quantitative assessment of the impact of earthquake-induced geohazards on natural landscapes in Jiuzhaigou Valley	Journal of mountain science
Shi, H., Shi, T.G., Han, F., Liu, Q., Wang, Z., Zhao, H.L.	2019	Conservation Value of World Natural Heritage Sites' Outstanding Universal Value via Multiple Techniques-Bogda, Xinjiang Tianshan	Sustainability
Santoro, A., Martinez, Aguilar, E.A., Venturi, M., Piras, F.	2020	The agroforestry heritage system of Sabana de Morro in El Salvador	Forests
Gordon, J.E., Crofts, R., Díaz-Martínez, E., Woo, K.S.	2018	Enhancing the role of geoconservation in protected area management and nature conservation	Geoheritage
Silva, F.M., Sousa, C., Albuquerque, H.	2022	Analytical Model for the Development Strategy of a Low-Density Territory: The Montesinho Natural Park	Sustainability
Zhang, M.H., Liu, J.Y.	2022	Does Agroforestry Correlate with the Sustainability of Agricultural Landscapes? Evidence from China's Nationally Important Agricultural Heritage Systems	Sustainability
Mavah, G.A., Funk, S.M., Child, B., Swisher, M.E., Nasi, R., Fa, J.E.	2018	Food and livelihoods in park-adjacent communities: The case of the Odzala Kokoua National Park	Biological Conservation
de Lima Marques, A., da Costa, C.R. G., Moura, D.C.	2019	ESPAÇOS DE CONFLITOS AMBIENTAIS DA ZONA DE AMORTECIMENTO DO PARQUE ESTADUAL MATA DO PAU FERRO (AREIA-PARAÍBA)	Geoambiente On-line
Vallejo,M.,Ramírez,M.I.,Reyes-González, A., López-Sánchez , J.G., Casas , A.	2019	Agroforestry systems of the Tehuacán-Cuicatlán Valley: Land use for biocultural diversity conservation	Land
Kuempel, C.D., Simmons, B.A., Davey, M.	2022	Assessing the status of existing and tentative marine World Heritage areas reveals opportunities to better achieve World Heritage Convention goals	Journal of Environmental Management
Kholilah, T.N., Rahimah, H., Hamzah, R., Arifianto, A.,Retnaningdyah, C., Hakim, L	2019	The sloping agricultural land management on buffer zone of Bromo Tengger Semeru National Park in Malang Regency	International Conference on Green Agro-Industry and Bioeconomy (ICGAB)
Jones, S.	2007	Tigers, trees and Tharu: An analysis of community forestry in the buffer zone of the Royal Chitwan National Park, Nepal	Geoforum

Table S1. List of the 128 publications considered in bibliometric analysis

Authors	Year	Name of article	Publication
Stapp, J.R., Lilieholm, R.J., Upadhaya,S., Johnson, T	2015	Evaluating the Impacts of Forest Management Policies and Community-Level Institutions in the Buffer Zone of Chitwan National Park, Nepal	Journal of sustainable forestry
Jiang, S.L., Xiong, K.N., Xiao, J.	2022	Structure and Stability of Agroforestry Ecosystems: Insights into the Improvement of Service Supply Capacity of Agroforestry Ecosystems under the Karst Rocky Desertification Control	Forests
Xiao, J., Xiong, K.N.	2022	A review of agroforestry ecosystem services and its enlightenment on the ecosystem improvement of rocky desertification control	Science of The Total Environment
Ayad, Y.M.	2005	Remote sensing and GIS in modeling visual landscape change: a case study of the northwestern arid coast of Egypt	Landscape and Urban Planning
Canedoli, C., Ficetola, G.F., Corengia, D., Tognini, P.	2022	Integrating landscape ecology and the assessment of ecosystem services in the study of karst areas	Landscape Ecology
Zhang, M., Xiong, K.N., Wang, X., Zhao, X.	2022	Natural Beauty and Esthetic Value of Natural World Heritage Sites: a literature Review and Implications for Karst Geoheritage Sites	Geoheritage
Zhang, J., Xion,g K.N., Liu, Z.J., He, L.X.	2022	Research progress on world natural heritage conservation: its buffer zones and the implications	Heritage Science
Moreno, G., Aviron, S., Berg, S., Crous-Duran, J., Franca, A., de Jalon, S.G., Hartel ,T., Mirck, J., Pantera, A., Palma, J.H.N.	2018	Agroforestry systems of high nature and cultural value in Europe: provision of commercial goods and other ecosystem services	Agroforestry System
Buck, L., Scherr, S., Trujillo, L., Mecham, J.	2020	Using integrated landscape management to scale agroforestry: examples from Ecuador	Sustainability Science
Attwater, R., Merson, J.	2007	Resilience of agri-industries neighbouring the Blue Mountains World Heritage Area	Systems Research and Behavioral Science
Hillbrand, A., Borelli, S., Conigliaro, M., Olivier, A.	2017	Agroforestry for landscape restoration: exploring the potential of agroforestry to enhance the sustainability and resilience of degraded landscapes	Food and Agriculture Organization of the United Nations
Santos, P., Crouzeilles, R., Sansevero, J.	2019	Can agroforestry systems enhance biodiversity and ecosystem service provision in agricultural landscapes?A meta-analysis for the Brazilian Atlantic Forest	Forest Ecology and Management
Santiago-Freijanes, J.J., Mosquera-Losada, M.R., Rois-Díaz, M., Ferreiro-Domínguez, N., Pantera, A., Vazquez, J.A.A., Rigueiro-Rodríguez, A.	2021	Global and European policies to foster agricultural sustainability: agroforestry	Agroforestry Systems

Table S1. List of the 128 publications considered in bibliometric analysis

Authors	Year	Name of article	Publication
Zou, Z., Zeng, F., Wang, K., Zeng , Z.X., Zhao, L.L., Du, H., Zhang, F., Zhang, H.	2019	Emergy and economic evaluation of seven typical agroforestry planting patterns in the karst region of Southwest China	Forests
Sun, Y., Cao, F., Wei, X., Welham, C., Chen, L., Pelz, D.R., Yang, Q., Liu, H.Q.	2017	An ecologically based system for sustainable agroforestry in sub-tropical and tropical forests	Forests
Anderson, S.H., Udawatta, R.P., Seobi, T., Garrett, H.E.	2009	Soil water content and infiltration in agroforestry buffer strips	Agroforestry systems
Bayala, J., Sanou, J., Teklehaimanot, Z., Kalinganire, A., Ouédraogo, S.J.	2014	Parklands for buffering climate risk and sustaining agricultural production in the Sahel of West Africa	Current Opinion in Environmental Sustainability
Dosskey,M.G.,Bentrup,G.,Schoeneberge, M.	2012	A role for agroforestry in forest restoration in the lower Mississippi alluvial valley	Journal of Forestry
Droppelmann, K., Berliner, P.	2003	Runoff agroforestry—a technique to secure the livelihood of pastoralists in the Middle East	Journal of Arid Environments
Epanda, M.A., Fotsing, A.J.M., Bacha, T., Frynta, D., Lens, L., Tchouamo, I.R., Jef, D.	2019	Linking local people's perception of wildlife and conservation to livelihood and poaching alleviation: A case study of the Dja biosphere reserve, Cameroon	Acta Oecologica
Gao, J., Barbieri, C., Valdivia, C.	2014	A socio-demographic examination of the perceived benefits of agroforestry	Agroforestry systems
Garrity, D.P.	1995	Buffer Zone Management and Agroforestry: Some lessons from a global perspective	Central Mindanao University, Museum, Bukidnon
Götmark, F., Söderlundh, H., Thorell, M.	2000	Buffer zones for forest reserves: opinions of land owners and conservation value of their forest around nature reserves in southern Sweden	Biodiversity & Conservation
Hansen, A.J., DeFries, R.	2007	Ecological mechanisms linking protected areas to surrounding lands	Ecological applications
Heredia-R, M., Torres, B., Cayambe, J., Ramos, N., Luna, M., Diaz-Ambrona, C.G.H.	2020	Sustainability Assessment of Smallholder Agroforestry Indigenous Farming in the Amazon: A Case Study of Ecuadorian Kichwas	Agronomy
Hjortsø, C.N., Stræde, S., Helles, F.	2006	Applying multi-criteria decision-making to protected areas and buffer zone management: A case study in the Royal Chitwan National Park, Nepal	Journal of forest economics
Kiyani, P., Andoh, J., Lee, Y., Lee, D.K.	2017	Benefits and challenges of agroforestry adoption: a case of Musebeya sector, Nyamagabe District in southern province of Rwanda	Forest science and technology
Masozera, M.K., Alavalapati, J.R.R.	2004	Forest dependency and its implications for protected areas management: a case study from the Nyungwe Forest Reserve, Rwanda	Scandinavian Journal of Forest Research

Table S1. List of the 128 publications considered in bibliometric analysis

Authors	Year	Name of article	Publication
Chen, X.Q.,Song, F.	2022	Integrity Evaluation of Natural Heritage: Development and Challenges	Study on Natural and Cultural Heritage
Ma, J.	2019	An Evaluation Model And Analysis With The Key Elements Of Impact In World Natural Heritage Sites Based On Remote Sensing	Wuhan University
Yang, J.	2018	Research on Impact of Reservoir Construction on World Natural Heritage——with a special reference to Bing'an Reservoir, Chishui, Guizhou	Guizhou Normal University
Yuan,Z.X., Liu, S.X.	2007	Legal Thinking on Maintaining the Authenticity and Integrity of World Natural Heritage -- Taking Wulingyuan as an Example	Northern Economy
Yuan, Z.X.	2006	Discussion on the Countermeasures of Maintaining the Authenticity and Integrity of World Natural Heritage -- Taking Wulingyuan as an Example	Chinese Public Administration
Zhang, C.Y.	2011	Authenticity and Integrity: Questioning and Rethinking	Southeast Culture
Wang, Y.L.	2010	Preliminary study about generation and development of world heritage integrity concept	Tsinghua University
Wu, L.Y.	2009	The Principle of Authenticity and Integrity and the Protection of World Heritage Resources in Mount Tai	Social Scientist
Zhang, C.Y.	2004	Interpretation and Analysis for 2 Important Conceptionsof the World Heritage Convention: Study on the World Heritage's Authenticity and Integrity	Acta Scientiarum Naturalium Universitatis Pekinensis
Ma, Y.W., Zhao, Y.T., Chen, F.B., Lan, L.B.	2011	Impact of Ya'an-Kangding highway on the world natural heritage of Sichuan giant panda sanctuaries	Resources and Environment in the Yangtze Basin
Zhang, C.Y.	2010	A Summary of Heritage Authenticity and Integrity Studies at Home and Abroad	Southeast Culture
Song, F., Zhu, J.j., Li, Y.F.	2009	Retrospection on the "Integrity" Principle of World Heritage——Review of the 4 Concepts of the Operational Guidelines for the Implementation of the World Heritage Convention	chinese landscape architecture
Zhang, S., Zhen, X.F.	2007	Assessment factors and social value of heritage protection integrity	Tongji University
Zhang, C.Y., Xie, N.G.	2003	The Principles of Authenticity and Integrity and the Conservation of the World Heritage	Journal of Peking University(Philosophy and Social Sciences)
Luo, X.	2022	Coupling mechanism and regulation of karst world heritage value protection and agroforestry development in buffer zone	Guizhou Normal University

Table S1. List of the 128 publications considered in bibliometric analysis

Authors	Year	Name of article	Publication
Xiong, K.N., Rong, L., Chen, H., Ying, B., Du, F.J., Xiao, S.Z, Liu, L.	2018	Value, Integrity, Protection and Management of Fanjing Mountain World Heritage	The New Era of Ecological Civilization
Xiong, K.N., Zhang, Z.Z., Xiao, S.Z., Di, Y.N., Xiao, H., Zhang, Y., Zhang, Y., Liu, S.X.	2020	Impact of Guinan Railway Construction on the Geomorphologic Value of the Libo-Huanjiang Karst World Heritage Site	Tropical Geography
Xiao, S.Z., Xiao, H., Wu, Y.H.	2020	Assessment of construction project on the aesthetic values of world heritage landscape based on GIS viewshed analysis: a case study of Wulingyuan World Natural Heritage Site	Journal of Guilin University of Technology
Bi, X.T., Han, F.	2018	Overview of Identification and Evaluation of Aesthetic Values of World Natural Heritage	Landscape Architecture
Zhong, Y.	2014	Global Comparative Analysis on Landscape Aesthetics and World Heritage Values of South China Karst	Guizhou Normal University
Li, G.C.	2014	Global Geomorphic Comparison and World Heritage Values of South China Karst	Guizhou Normal University
Wang, X.	2020	Study on the Function of Buffer Zone in World Heritage Protection -- Taking Liangzhu Site as an Example	China Cultural Heritage
Jia, L.Q., Guo, H.M.	2015	Revision and Interpretation of the "Buffer Zone" Clause in the Operational Guidelines for the Implementation of the World Heritage Convention	Planners
Jia, L.Q.	2015	Research on the planning and implementation mechanism of the World Heritage buffer zone from the Perspective of the Scenic Area in China	Tsinghua University
Gao, J.J.	2018	Heritage Protection and Landscape Renewal of Beijing Section of the Beijing Hangzhou Grand Canal -- Definition of Buffer Zone	Juse
Wen, S.P.	2009	On the Integrity of World Heritage -- A Case Study of Shenyang Forbidden City	Theory Horizon
Sun, Y.	2018	A Brief Analysis of the Integrity Assessment and Protection of World Cultural Heritage from the Perspective of Visual Integrity	China Cultural Heritage
Zhou, N.X., Lin, Z.S., Huang, Z., Fang, P.G.	2008	Threats on the world heritage natural sites and China's conservation countermeasures	Journal of Natural Resources
Zhang, C.Z.	2007	Indicators for World Heritage Governance Evaluation and Its Application	Journal of Central South University of Forestry & Technology(Social Sciences)
Pang, X.C.	2021	Discussion on the development of agro forestry in nature reserves and surrounding communities	South China Agriculture
Hu C.D., Chen, Z.R., Jia, L.Z., Tian, L.H.	2009	Discussion on mixed agriculture and forestry in communities around nature reserves	Modern Agricultural Science and Technology

Table S1. List of the 128 publications considered in bibliometric analysis

Authors	Year	Name of article	Publication
Zhao, Z.C., Yang, R.	2021	The concept of national park authenticity and integrity in China and its evaluation framework	Biodiversity Science
Xie, Y., Du, J., Pei, X.j., Zou, Z., Tang, Y., Wang, Y., Wang, X., Yang, Q.X., Qiao, X.	2021	Ecological Rehabilitation of Natural World Heritage Based on Outstanding Universal Value—A Case Study on Jiuzhaigou's Sparkling Lake	Study on Natural and Cultural Heritage
Han, X., Hu, J.L., Liu, C.S., Wang, X.Y.	2021	Mountain landscape health diagnosis and pattern evolution based on remote sensing: Case study in Xinjiang Tianshan Karajun-Kurdening	Acta Ecologica Sinica
Leng, Z.M., Ma, X.J.	2009	Protection and utilization of china's world natural heritages	Economic Geography
Fu, J., Li, C., Wang, M., Yan J.Q.	2014	Overall characteristic protection strategies of slender west lake based on the "completeness"concept	China Ancient City
Zhang, P.Q., Zhou, C.X., Bai, J.D., Li, X.S., Lin, D.Y.	2020	Preliminary study on the relationship between protected area and world heritage site	Study on Natural and Cultural Heritage
Lu, X.S., Huang, D.L.	2006	About the concept of natural heritage and some related issues	Journal of Hunan Ecological Science
Wang, Y.L., Zhuang, Y.B., Li, J.H.	2014	Study and reference on the protection and management planning in buffer zone of protected areas in Nepal	Urban Planning International
Wang, Y.L., Li, X.L., Liu, C.Q.	2014	A prove regarding Sanqingshan world natural heritage sustainable development	Chinese Landscape Architecture
Yu, K.J., Li, B., Li, D.H.	2008	Ecological Infrastructure Approaches to the Protection of Natural and Cultural Heritage Areas: A Case Study of Wuyi Mountain in Fujian Province	City Planning Review
Wu, Q.L., Liang, H., Xiong, K.N., Li, R.	2018	Frontier Theories and Counter measures for Integrated Regulation of Soil and Water Loss and Mountainous Agroforestry in Rocky Desertification Environment.	Journal of Soil and Water Conservation
Xiong, K.N., Xiao, ., Zhu, D.Y.	2022	Progress of research on agroforestry ecosystem services and implications for industrial revitalizati	Acta Ecologica Sinica
Zhang, L.B., Wang, W., Wu, C.X., Xiong, Y.J.	2008	Quantitative method of visual landscape EIA based on GIS:a case of the Ming Tombs	Acta Ecologica Sinica
Chen, P.D., Xiao, S.Z.	2019	Study on Landscape Aesthetic Characteristics and Value Improvement of Shibing World Natural Heritage Site	China Place Name
Zhang, M.R., Zhai, M.P., Yin, C.J., Wang, X.Y.	2003	Progress on the study of soil erosion by using isotopes as tracer	Science of Soil and Water Conservation
Liu, X.G.	2005	On protection of Wulingyuan world natural heritage	Engineering Construction

Table S1. List of the 128 publications considered in bibliometric analysis

Authors	Year	Name of article	Publication
Sun, K.Q.	2010	Issues and approaches of world natural heritage site of South China Karst	Resource Development & Market
Yang, Q., Zhou, Z.F., Liu, M.Q.	2009	Soil Erosion Remote Monitoring of World Natural Heritage Site and Conservation of the Heritage Site—Example for the Libo World Natural Heritage Site in Guizhou	Journal of Liupanshui Normal University
Yang, Z.P., Xu, X.L., Ding, X.Q., Han, F., Zhang, Y.M., Yang, W.K.	2012	Study on the value, protection and management of natural heritage in tianshan mountain, xinjiang	Xinjiang Institute Of Ecology And Geography，Chinese Academy Of Sciences
Yang, Q., Zhou, Z.F.	2007	3S technology and scientific discussion of boundary identification of nominated site for the world natural heritage - A case study of the Libo nominated site for "the South China Karst"	Journal of Guizhou Normal University(Natural Sciences)
He, F.Y., Xiong, K.N., Zhu, D.Y.	2020	Advances in research on water effects of agroforestry in karst mountains	China Feed
Qin, D.J., An, H.P.,Dai, Z.F.	2003	Management Mode and Benefit Analysis of Agroforestry in Guizhou Karst Region -- A Case Study of Southwest Guizhou	Guizhou Forestry Science and Technology
Wang, Q.Y., Ma, Y.S.	2014	Research on community participation mechanism in natural heritage conservation and development	Journal of Jiangxi University of Science and Technology
Zhou, X.Y., Li, J.H., Yang, H.K., Zhang, X.Y., Wang, M.N., Cao, S.Y.	2022	Study on the potential of world natural heritage in Xingkai Hu nature reserve,Heilongjiang Province	Study on Natural and Cultural Heritage
Zhuang, Y.B.	2013	Overview of management planning practice for Chinese world natural heritage sites	Chinese Landscape Architecture
Han, X., Liu, C.S., Hu, J.L., Wang, X.Y., Luo, L., Zhao, Y.C., Li, L., Ji, X.Y., Yan, H., Wang, Y.	2019	Dynamic evolution of landscape pattern and ecological health assessment of Tianshan natural heritage site in Xinjiang	Arid Land Geography
Zhao, Y., Luo, L., Wan, H., Wu, Q., Liu, C.S.	2021	Remote sensing monitoring and analysis of landscape pattern in Bayanbulak Heritage Site for nearly 30 years	National Remote Sensing Bulletin
